# The longitudinal association between coffee and tea consumption and the risk of metabolic syndrome and its component conditions in an older adult population

**DOI:** 10.1017/jns.2022.78

**Published:** 2022-09-21

**Authors:** Tommy Hon Ting Wong, George Burlutsky, Bamini Gopinath, Victoria M. Flood, Paul Mitchell, Jimmy Chun Yu Louie

**Affiliations:** 1 School of Biological Sciences, The University of Hong Kong, Pokfulam, Hong Kong SAR; 2 Centre for Vision Research, The Westmead Institute for Medical Research, The University of Sydney, Sydney, NSW, Australia; 3 Department of Linguistics, Macquarie University, Sydney, NSW, Australia; 4 University Department of Rural Health, Northern Rivers; School of Health Sciences, Faculty of Medicine and Health, The University of Sydney, Lismore, NSW, Australia; 5 Westmead Hospital, Western Sydney Local Health District, Westmead, Sydney, NSW, Australia

**Keywords:** Coffee, Longitudinal study, Metabolic syndrome, Older adults, Tea

## Abstract

The present study aimed to assess the longitudinal associations of coffee and tea consumption with metabolic syndrome and its component conditions in a group of Australian older adults who participated in the Blue Mountains Eye Study (*n* 2554, mean age: 64 years, 43 % female). Participants’ coffee and tea intake were measured using a validated food frequency questionnaire. Hazard ratios (HRs) over a 10-year period were estimated using Cox hazard regression models adjusting for lifestyle factors. Results showed that coffee consumption was not associated with the incidence of metabolic syndrome, high fasting glucose, high triglycerides, central obesity, high blood pressure and low HDL-cholesterol (HDL-C). Tea consumption was not associated with incidence of metabolic syndrome and the component conditions except for the risk of having low HDL-C, in which a nominally inverse association was observed (multivariate-adjusted HR at 2–3 cups/d: 0⋅48, 95 % CI 0⋅26, 0⋅87, *P* = 0⋅016; 4 cups/d or more: 0⋅50, 95 % CI 0⋅27, 0⋅93, *P* = 0⋅029). After stratifying for fruit consumption (*P*_interaction_ between tea and fruit = 0⋅007), consuming four cups of tea per day was nominally associated with lower incidence of metabolic syndrome among those with high fruit consumption (multivariable-adjusted HR: 0⋅44, 95 % CI 0⋅20, 0⋅93, *P* = 0⋅033). Our results did not support a significant association between tea and coffee consumption and metabolic syndrome. Tea consumption may be associated with a lower risk of having low HDL-C, while high tea and fruit consumption together may be associated with a lower risk of developing metabolic syndrome.

## Introduction

Metabolic syndrome is defined as the co-occurrence of several metabolic anomalies, including central obesity, high fasting glucose levels, high triglycerides levels, low high-density-lipoprotein-cholesterol (HDL-C) levels and high blood pressure (BP)^(1)^. Patients with metabolic syndrome were two- and five-times as likely to develop cardiovascular diseases (CVD) and type 2 diabetes mellitus (T2DM), respectively, when compared with those without this condition^(2)^. Metabolic syndrome is estimated to be affecting 20–30 % of the population around the globe^(3–6)^ and its prevalence was found to be higher in elderly population^(7,8)^, possibly due to the age-related decline in homeostatic regulation and diminishing anti-inflammatory mechanisms^(9)^.

Both coffee and tea consumption have been inversely associated with metabolic syndrome^(10)^ and one proposed explanation was the rich content of polyphenols. Both tea and coffee are major sources of dietary polyphenols, such as chlorogenic acids and caffeic acids in coffee, as well as catechins and gallic acid in tea. These polyphenols were shown to improve oxidative stress and insulin resistance^(11,12)^, both of which were important mediators in the development of metabolic syndrome^(1)^.

However, the significant associations between coffee consumption and metabolic syndrome, as well as its component conditions, were only observed in cross-sectional studies^(13)^, leading to worries that such results could be biased by residual confounding and reverse causation. In support of this statement, longitudinal studies in this regard have only been conducted in younger populations^(14–16)^ and did not yield any significant findings, while the longitudinal association between tea and coffee consumption and the incidence of metabolic syndrome has not been previously investigated in an elderly population.

We hereby report the findings from analysing the data of the Blue Mountain Eye Study (BMES), which is an Australian elderly cohort followed for 10 years with dietary intake and disease outcome recorded. The primary aim of the present study is to investigate the prospective associations between tea and coffee consumption and the risk of developing metabolic syndrome. The secondary aims included (1) to investigate the prospective associations between tea and coffee consumption and the risks of developing the component conditions of metabolic syndrome, including high fasting glucose levels, high triglycerides levels, low HDL-C, central obesity and high BP, and (2) to investigate the effect modification due to the confounding variables in the associations tested.

## Method

### Study population

The BMES collected data related to vision, diet and lifestyle factors from people in the areas of Katoomba, Leura and Wentworth Falls in the west of Sydney, New South Wales, Australia. Details of the BMES have been previously reported^(17,18)^. In brief, non-institutionalized residents in these areas who were born before 1 January 1943 (i.e. 49 years old and over) were invited to participate. The baseline assessment (BMES I) was conducted during 1992–4 and eligible residents were invited to carry out an eye examination at a local clinic, where they also provided data in anthropometric measures, lifestyle factors and dietary intake. Participants of BMES I were then invited to attend follow-up surveys 5 (BMES II, 1997–9) and 10 years later (BMES III, 2002–4), during which data regarding anthropometric measurements, regarding lifestyle factors and dietary intake were collected. Participants also provided fasting blood samples after an overnight fast for measuring biomarkers at baseline and at each follow-up.

**Fig. 1.**
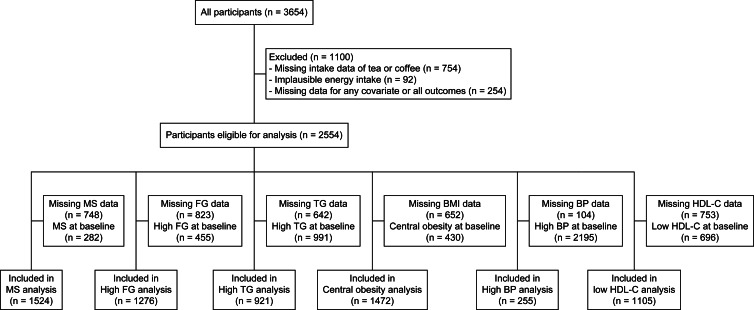
Participant inclusion flowchart of all analyses. The sum of participants included for all outcomes does not equal the total number of participants included in this analysis as some provided data for multiple outcomes. MS, metabolic syndrome; FG, fasting glucose; TG, triglycerides; BP, blood pressure; BMI, body mass index; HDL-C, high-density-lipoprotein-cholesterol.

The exclusion criteria are as follows: missing data in coffee or tea consumption, with implausible energy intake (female: <500 kcal/d or >3500 kcal/d; male: <800 kcal/d or >4000 kcal/d), missing data in any of the covariates, or missing data for all outcome variables. In this analysis, participants lost to follow up were censored at their previous attendance^(19)^, thus those who were lost to follow-up at BMES III were treated as censored at BMES II, while those who did not provide data at BMES II were excluded from the analysis. Furthermore, participants meeting the criteria of the conditions at baseline were excluded from the corresponding analyses. The participant flowchart for all outcomes is shown in Fig. 1. From the total of 3654 BMES participants recruited at baseline, 2554 provided data for at least one outcome in this analysis.

### Coffee and tea consumption data collection

Prior to the participants’ clinic visits in each survey, they were sent a 145-item semi-quantitative food frequency questionnaire (FFQ), which was modified from an early FFQ by Willet *et al.*^(20)^ for assessing their dietary intake in the year prior. Participants reported their usual frequency of food intake at a given serve size using a 9-category frequency scale with the following responses: never, less than 1 per month, 1–3 per month, 1 per week, 2–4 per week, 5–6 per week, 1 per day, 2–3 per day and 4+ per day. The BMES FFQ was validated against three separate 4-d weighed food records in a randomly selected subsample of the BMES cohort^(21)^. Spearman's correlations were above 0⋅5 for most nutrients when compared with the results of the weighed food records. The short-term (4–6 weeks) and long-term reproducibility (12–18 months) were also found to be satisfactory – correlations ranged from 0⋅6 to 0⋅8 for most nutrients. Consumption frequencies of caffeinated coffee, decaffeinated coffee and tea were asked in separate questions, all in the serve size of one cup (250 ml). Participants also reported coffee and tea consumption, along with other dietary intake, in the follow-up surveys. Responses for coffee consumption were consolidated into three groups: less than 1 cup per week, 1 cup per week to 1 cup per day and 2 cups per day or above. Responses of tea consumption were consolidated into three groups: 1 cup per day or less, 2–3 cups per day and 4 cups per day or above.

### Metabolic syndrome ascertainment

The definition proposed by the International Diabetes Federation in 2006^(22)^ was used to define metabolic syndrome, which was diagnosed by having central obesity, plus any two of the following conditions: high fasting glucose levels, high triglycerides levels, high blood pressure or low HDL-C levels. Central obesity was defined as body mass index (BMI) ≥30 kg/m^2^. It is shown in Australian data that central obesity can be assumed without waist circumference measurement if a person's BMI is higher than 30 kg/m^2(2)^. High triglyceride level was defined as ≥1⋅7 mmol/l. High fasting glucose was defined as ≥5⋅6 mmol/l, or with T2DM (defined as self-reported physician diagnosis of T2DM plus taking medication for T2DM or fasting glucose ≥7⋅0 mmol/l). High blood pressure was defined as systolic pressure ≥130 mmHg, or diastolic pressure ≥85 mmHg, or on medication for treating high BP. Low HDL-C level was defined <1⋅03 mmol/l in males or <1⋅29 mmol/l in females.

At the clinic visit, weight was assessed using digital scales with participants wearing light clothes and no shoes. Height was assessed using a stadiometer and BMI was calculated in kg/m^2^. Seated BP was measured using standard auscultatory methods and a mercury sphygmomanometer. At the end of the visit, participants were asked to return on a morning within 4 weeks to provide fasting blood samples, which were collected in fluoride and oxalate tubes. All samples were centrifuged on-site within 1 h to separate plasma. Samples were then refrigerated before being transported on ice to Westmead Hospital, Sydney, for biochemical analysis. Glucose levels were measured by the hexokinase method. HDL-C and triglycerides were measured using an automatic biochemistry analyzer (Hitachi 747 Biochemistry Analyzer, Tokyo, Japan).

### Data collection of confounding variables

The following factors were previously found to be associated with metabolic syndrome and were identified as confounding variables in this analysis: smoking habit^(23)^, physical activity level^(24)^, socioeconomic status^(25)^, family history of diabetes^(26)^, intake of fruit and vegetables^(27)^, dairy products^(28)^ and alcohol^(29)^. Smoking status at baseline, education level and family history of T2DM (with or without a family history of T2DM) were self-reported. Physical activity was quantified in terms of metabolic equivalents (METs) based on the International Physical Activity Questionnaire scoring protocol^(30)^, which was done by analysing the participants’ reported performance, duration and frequency of walking and exercises in the past 2 weeks. Intake of food items in the FFQ was categorised into major food groups based on descriptors and macronutrient content^(31)^. Nutrient intake was estimated using the Australian Tables of Food Composition 1990^(32)^.

### Statistical analysis

The primary exposures were coffee and tea consumption frequency at baseline, while the primary outcome was the incidence of metabolic syndrome, while the secondary outcomes were the incidence of high fasting glucose levels, high triglycerides levels, high BP, central obesity and low HDL-C levels. The Cox proportional hazard regression was used to estimate the hazard ratios (HRs) of participants in different consumption groups having metabolic syndrome, as well as the component conditions, when compared with those in the lowest consumption category. The proportional hazard assumption was checked for each covariate by inspecting the Schoenfeld residuals in each exposure-outcome pair. It was found that the vegetable intake variable violated the proportional hazard assumption in the association between coffee and high triglyceride level, as well as that between tea and high triglyceride level. As a result, vegetable intake was replaced by the interaction term of vegetable intake and time as a covariate in the analyses of both associations.

Three models were used to estimate the HRs for each outcome variable. For analyses using coffee consumption frequency as the predictor, model 1 was adjusted for baseline age and sex. Model 2 further adjusted for baseline smoking status (never smoked/previous smoker/current smoker), METs categories (zero/low/high), whether a qualification was obtained after leaving school (yes/no), family history of T2DM (yes/no) and the following dietary intake data collected at baseline: total energy intake, intake of vegetables, fruits, dairy food and alcohol consumption categories (zero/low/high). Model 3 further adjusted for tea consumption frequency. For analyses using tea consumption frequency as the predictor, covariates in models 1 and 2 were the same as those in coffee consumption analysis, while model 3 was further adjusted for coffee consumption frequency. The *P* values for trends were obtained by using the median values in each consumption group as the exposure variables in the Cox proportional hazard models.

Statistical significance for all associations was set at *P* < 0⋅0083 (Bonferroni correction: 0⋅05 divided by 6 outcomes). All analyses were done using the ‘survival’ package^(33)^ in R software version 4.0.3^(34)^.

### Sensitivity analyses

The effect of changes in coffee or tea consumption was tested by repeating the main analysis using tea and coffee consumption averaged over all follow-ups. To exclude the effect possibly from decaffeinated coffee intake, we repeated the main analysis after excluding participants reported consuming decaffeinated coffee at baseline. In addition, we also assessed the association between coffee intake and metabolic syndrome in tea abstainers, as well as the association between tea intake and metabolic syndrome in coffee abstainers only.

Extra analyses were also conducted to investigate whether the associations were modified by confounding variables, as done in previous longitudinal studies^(35,36)^. The *P* values of the interaction term between the exposure variable (i.e. coffee or tea consumption) and the covariates were obtained from likelihood ratio *χ*^2^ tests of the Cox proportional hazard models with and without the interaction term. When a significant interaction (*P* < 0⋅0083) was found, the analysis was stratified by the covariate. If the variable is continuous, stratification was conducted by a median split.

## Results

### Associations between coffee consumption and metabolic syndrome and the component conditions

The characteristics of the included participants, stratified by coffee consumption frequencies, are shown in Table 1. When compared with participants who consumed coffee less than once per week, those who consumed coffee more frequently were less likely to be males, more likely to be current smokers, had higher energy and alcohol intakes, and consumed less tea. The characteristics of participants that were loss to follow-up were similar to those of the included participants (Supplementary Tables S1–S6).

**Table 1.** Baseline characteristics of participants included in the analysis of metabolic syndrome, stratified by coffee consumption frequencies (*n* 2554)^a^

	Less than 1 cup/week (*n* 725)	1 cup/week – 1 cup/d (*n* 822)	2 cups/d or above (*n* 1007)
Age (years)	67⋅0 ± 9⋅4	65⋅8 ± 9⋅2	63⋅4 ± 8⋅6
Male (%)	61⋅5	56⋅6	54⋅4
Female (%)	38⋅5	43⋅4	45⋅6
Non-smoker (%)	55⋅0	52⋅4	41⋅6
Former smoker (%)	35⋅6	37⋅3	39⋅1
Current smoker (%)	9⋅4	10⋅2	19⋅3
Obtained qualification after leaving school (%)	43⋅6	34⋅5	41⋅2
METs categories (%)			
Zero METs	29⋅2	25⋅2	23⋅6
Low METs	35⋅3	39⋅1	38⋅0
High METs	35⋅4	35⋅8	38⋅3
With family history of T2DM (%)	23⋅0	20⋅8	22⋅6
Energy (kJ)	8146 ± 2240	8530 ± 2219	8472 ± 2240
Alcohol consumption categories (%)^b^			
Zero	33⋅2	18⋅1	14⋅7
Low	41⋅0	37⋅6	39⋅5
High	25⋅8	44⋅3	45⋅8
Vegetables (g)	451 ± 195	442 ± 185	426 ± 178
Fruit (g)	344 ± 248	343 ± 242	337 ± 251
Dairy (g)	347 ± 267	336 ± 236	347 ± 252
Tea consumption categories (%)			
1 cup/d or less	27⋅4	29⋅0	50⋅0
2–3 cups/d	29⋅9	36⋅1	34⋅5
4 cups/d or above	42⋅6	34⋅9	15⋅6

a Values are mean ± sd or percentages. METs, metabolic equivalents; T2DM, type 2 diabetes mellitus.

b Participants in the ‘zero’ category reported zero alcohol consumption. The mean ± sd of alcohol consumption in the low and high categories were 2 ± 2 g and 25 ± 18 g, respectively.

The results of Cox regressions are shown in Table 2. The overall incidence of metabolic syndrome was around 8 % after 10 years of follow-up. After adjusting for all covariates, no remarkable associations were observed between coffee consumption and metabolic syndrome (multivariate-adjusted HR of 1 cup/week to 1 cup/d: 1⋅08, 95 % CI 0⋅66, 1⋅75, *P* = 0⋅760; 2 cups/d or more: 1⋅16, 95 % CI 0⋅73, 1⋅84, *P* = 0⋅535) and no significant trends were observed. The associations with high fasting glucose, high triglycerides, central obesity, high blood pressure and low HDL-C were near null. Assessing average coffee intake as a continuous variable did not yield statistically significant results in any analysis (result not shown).

**Table 2.** Associations between coffee consumption frequency and incidence of metabolic syndrome, high fasting glucose, high triglycerides, high blood pressure, obesity and low HDL-C^a^

	Less than 1 cup/week	1 cup/week – 1 cup/d		2 cups/d or more		
	HR	HR (95 % CI)	*P*^b^	HR (95 % CI)	*P*^b^	*P*_trend_^c^
Metabolic syndrome						
No. of case/total no. of participants, *n*/total *n*	30/400	41/510		58/614		
Model 1	1 (ref)	1⋅07 (0⋅67, 1⋅72)	0⋅778	1⋅18 (0⋅76, 1⋅84)	0⋅460	0⋅441
Model 2	1 (ref)	1⋅08 (0⋅66, 1⋅74)	0⋅769	1⋅16 (0⋅74, 1⋅83)	0⋅512	0⋅502
Model 3	1 (ref)	1⋅08 (0⋅66, 1⋅75)	0⋅760	1⋅16 (0⋅73, 1⋅84)	0⋅535	0⋅532
High fasting glucose level						
No. of case/total no. of participants, *n*/total *n*	73/339	79/428		106/509		
Model 1	1 (ref)	0⋅84 (0⋅61, 1⋅15)	0⋅282	0⋅97 (0⋅72, 1⋅31)	0⋅856	0⋅934
Model 2	1 (ref)	0⋅83 (0⋅60, 1⋅16)	0⋅278	0⋅92 (0⋅67, 1⋅26)	0⋅610	0⋅806
Model 3	1 (ref)	0⋅81 (0⋅58, 1⋅12)	0⋅204	0⋅85 (0⋅61, 1⋅17)	0⋅309	0⋅439
High triglycerides^d^						
No. of case/total no. of participants, *n*/total *n*	39/226	54/319		64/376		
Model 1	1 (ref)	1⋅01 (0⋅67, 1⋅53)	0⋅948	0⋅92 (0⋅62, 1⋅37)	0⋅682	0⋅620
Model 2	1 (ref)	1⋅10 (0⋅72, 1⋅69)	0⋅668	0⋅91 (0⋅60, 1⋅38)	0⋅646	0⋅49
Model 3	1 (ref)	1⋅11 (0⋅72, 1⋅71)	0⋅634	0⋅96 (0⋅62, 1⋅48)	0⋅855	0⋅719
Central obesity						
No. of case/total no. of participants, *n*/total *n*	53/392	64/493		79/587		
Model 1	1 (ref)	0⋅94 (0⋅65, 1⋅35)	0⋅718	0⋅92 (0⋅65, 1⋅31)	0⋅661	0⋅693
Model 2	1 (ref)	0⋅86 (0⋅59, 1⋅24)	0⋅412	0⋅88 (0⋅61, 1⋅26)	0⋅477	0⋅579
Model 3	1 (ref)	0⋅85 (0⋅59, 1⋅24)	0⋅400	0⋅88 (0⋅61, 1⋅27)	0⋅496	0⋅600
High blood pressure						
No. of case/total no. of participants, *n*/total *n*	59/72	50/63		97/120		
Model 1	1 (ref)	0⋅98 (0⋅67, 1⋅43)	0⋅924	0⋅97 (0⋅70, 1⋅34)	0⋅834	0⋅834
Model 2	1 (ref)	0⋅93 (0⋅62, 1⋅40)	0⋅742	0⋅97 (0⋅68, 1⋅37)	0⋅860	0⋅920
Model 3	1 (ref)	0⋅96 (0⋅64, 1⋅44)	0⋅837	1⋅00 (0⋅70, 1⋅43)	0⋅979	0⋅932
Low HDL-cholesterol						
No. of case/total no. of participants, *n*/total *n*	21/297	19/367		32/441		
Model 1	1 (ref)	0⋅64 (0⋅34, 1⋅19)	0⋅162	0⋅87 (0⋅50, 1⋅52)	0⋅631	0⋅894
Model 2	1 (ref)	0⋅74 (0⋅39, 1⋅41)	0⋅361	0⋅90 (0⋅51, 1⋅60)	0⋅722	0⋅889
Model 3	1 (ref)	0⋅80 (0⋅42, 1⋅53)	0⋅500	0⋅83 (0⋅46, 1⋅50)	0⋅548	0⋅623

a Model 1 adjusted for baseline age and sex. Model 2 further adjusted for smoker status (non-smoker/former smoker/current smoker), metabolic equivalents categories (zero/low/high), qualification obtained after leaving school (yes/no), family history of type 2 diabetes mellitus (yes/no), total energy intake, intake of vegetables, fruits, dairy food and alcohol consumption categories (zero/low/high). Model 3 further adjusted for tea consumption. HDL, high-density-lipoprotein.

b *P* value associated with the HR.

c *P*_trend_ was obtained by using the median values of each consumption group as the exposure variables in the Cox proportional hazard models.

d Due to the violation of proportional hazard assumption, vegetable intake was replaced by the interaction term between vegetable intake and time in models 2 and 3.

### Associations between tea consumption and metabolic syndrome and the component conditions

The characteristics of the participants stratified by tea consumption frequencies are shown in Table 3. Those who consumed tea two times or more per day were more likely to be males and less likely to be current smokers. They also had a higher mean energy intake and consumed less coffee and alcohol. The characteristics of participants that were loss to follow-up were similar to those of the included participants (Supplementary Tables S7–S12).

**Table 3.** Baseline characteristics of participants included in the analysis of metabolic syndrome, stratified by tea consumption frequencies (*n* 2554)^a^

	1 cup/d or less (*n* 940)	2–3 cups/d (*n* 861)	4 cups/d or above (*n* 753)
Age (years)	63⋅8 ± 9⋅0	66⋅2 ± 9⋅5	65⋅7 ± 8⋅9
Male (%)	54⋅4	56⋅1	61⋅8
Female (%)	45⋅6	43⋅9	38⋅2
Non-smoker (%)	45⋅3	50⋅5	51⋅5
Former smoker (%)	36⋅8	39⋅1	36⋅7
Current smoker (%)	17⋅9	10⋅3	11⋅8
Obtained qualification after leaving school (%)	41⋅3	37⋅2	40⋅8
METs categories (%)			
Zero METs	26⋅6	25⋅0	25⋅5
Low METs	34⋅6	37⋅5	41⋅4
High METs	38⋅8	37⋅5	33⋅1
With family history of T2DM (%)	20⋅3	21⋅4	25⋅4
Energy (kJ)	8033 ± 2257	8543 ± 2207	8689 ± 2190
Alcohol consumption categories (%)^b^			
Zero	20⋅1	17⋅0	27⋅0
Low	36⋅9	40⋅4	41⋅0
High	43⋅0	42⋅6	32⋅0
Vegetables (g)	416 ± 187	449 ± 187	453 ± 178
Fruit (g)	340 ± 260	347 ± 240	335 ± 239
Dairy (g)	304 ± 244	349 ± 248	386 ± 258
Coffee consumption categories (%)			
<1 cup/week	21⋅2	25⋅2	41⋅0
1 cup/week – 1 cup/d	25⋅3	34⋅5	38⋅1
2 cups/d or above	53⋅5	40⋅3	20⋅8

a Values were mean ± sd or percentages. METs, metabolic equivalents; T2DM, type 2 diabetes mellitus.

b Participants in the ‘zero’ category reported zero alcohol consumption. The mean ± sd of alcohol consumption in the low and high categories were 2 ± 2 g and 25 ± 18 g, respectively.

The results of Cox regressions are shown in Table 4. The incidence of metabolic syndrome across the tea consumption frequencies ranged from 8 to 9 %. After adjusting for all covariates, no remarkable association was observed between tea consumption and metabolic syndrome (multivariate-adjusted HR of 2–3 cups/d: 0⋅93, 95 % CI 0⋅60, 1⋅42, *P* = 0⋅727; 4 cups/d or more: 0⋅95, 95 % CI 0⋅61, 1⋅48, *P* = 0⋅824). The associations between tea consumption and the incidence of high fasting glucose levels, high triglycerides levels, central obesity and high BP were not clinically and statistically significant and no significant trends were observed. Tea consumption at 2 cups/d or above was significantly associated with lower incidence of low HDL-C after adjusting for age and sex. The inverse association remained after adjusting for other lifestyle factors, yet it was no longer statistically significant after taking into account the Bonferroni correction factor (multivariate-adjusted HR at 2–3 cups/d: 0⋅48, 95 % CI 0⋅26, 0⋅87, *P* = 0⋅016; 4 cups/d or more: 0⋅50, 95 % CI 0⋅27, 0⋅93, *P* = 0⋅029). Assessing average tea intake as a continuous variable did not yield any significant results (result not shown).

**Table 4.** Associations between tea consumption and the incidence of metabolic syndrome, high fasting glucose, high triglycerides, high blood pressure, central obesity and low HDL-C^a^

	1 cup/d or less	2–3 cups/d		4 cups/d or more		
	HR	HR (95 % CI)	*P*^b^	HR (95 % CI)	*P*^b^	*P*_trend_^c^
Metabolic syndrome						
No. of case/total no. of participants, *n*/total *n*	48/533	41/520		40/471		
Model 1	1 (ref)	0⋅95 (0⋅62, 1⋅44)	0⋅799	0⋅95 (0⋅62, 1⋅44)	0⋅801	0⋅783
Model 2	1 (ref)	0⋅92 (0⋅60, 1⋅41)	0⋅712	0⋅92 (0⋅60, 1⋅42)	0⋅715	0⋅692
Model 3	1 (ref)	0⋅93 (0⋅60, 1⋅42)	0⋅727	0⋅95 (0⋅61, 1⋅48)	0⋅824	0⋅789
High fasting glucose level						
No. of case/total no. of participants, *n*/total *n*	97/439	92/442		69/395		
Model 1	1 (ref)	0⋅92 (0⋅69, 1⋅23)	0⋅569	0⋅75 (0⋅55, 1⋅03)	0⋅073	0⋅086
Model 2	1 (ref)	0⋅94 (0⋅70, 1⋅26)	0⋅682	0⋅75 (0⋅54, 1⋅03)	0⋅072	0⋅092
Model 3	1 (ref)	0⋅95 (0⋅70, 1⋅27)	0⋅716	0⋅73 (0⋅52, 1⋅01)	0⋅057	0⋅080
High triglycerides^d^						
No. of case/total no. of participants, *n*/total *n*	56/322	48/324		53/275		
Model 1	1 (ref)	0⋅93 (0⋅63, 1⋅36)	0⋅697	1⋅18 (0⋅81, 1⋅72)	0⋅390	0⋅482
Model 2	1 (ref)	1⋅07 (0⋅72, 1⋅60)	0⋅742	1⋅27 (0⋅86, 1⋅88)	0⋅222	0⋅245
Model 3	1 (ref)	1⋅05 (0⋅70, 1⋅57)	0⋅809	1⋅23 (0⋅82, 1⋅85)	0⋅307	0⋅337
Central obesity						
No. of case/total no. of participants, *n*/total *n*	65/511	69/508		62/453		
Model 1	1 (ref)	1⋅15 (0⋅82, 1⋅61)	0⋅431	1⋅08 (0⋅76, 1⋅53)	0⋅668	0⋅602
Model 2	1 (ref)	1⋅08 (0⋅77, 1⋅53)	0⋅649	1⋅07 (0⋅75, 1⋅52)	0⋅727	0⋅696
Model 3	1 (ref)	1⋅09 (0⋅77, 1⋅54)	0⋅628	1⋅05 (0⋅73, 1⋅52)	0⋅775	0⋅731
High blood pressure						
No. of case/total no. of participants, *n*/total *n*	91/108	67/91		48/56		
Model 1	1 (ref)	0⋅83 (0⋅60, 1⋅14)	0⋅242	1⋅14 (0⋅80, 1⋅62)	0⋅468	0⋅918
Model 2	1 (ref)	0⋅81 (0⋅58, 1⋅13)	0⋅222	1⋅08 (0⋅75, 1⋅56)	0⋅688	0⋅900
Model 3	1 (ref)	0⋅82 (0⋅58, 1⋅14)	0⋅234	1⋅09 (0⋅74, 1⋅59)	0⋅666	0⋅911
Low HDL-cholesterol						
No. of case/total no. of participants, *n*/total *n*	41/385	16/377		15/343		
Model 1	1 (ref)	0⋅43 (0⋅24, 0⋅77)	0⋅005	0⋅48 (0⋅27, 0⋅88)	0⋅017	0⋅003
Model 2	1 (ref)	0⋅47 (0⋅26, 0⋅85)	0⋅013	0⋅50 (0⋅27, 0⋅93)	0⋅028	0⋅008
Model 3	1 (ref)	0⋅48 (0⋅26, 0⋅87)	0⋅016	0⋅50 (0⋅27, 0⋅93)	0⋅029	0⋅009

a Model 1 adjusted for baseline age and sex. Model 2 further adjusted for smoker status (non-smoker/former smoker/current smoker), metabolic equivalents categories (zero/low/high), qualification obtained after leaving school (yes/no), family history of type 2 diabetes mellitus (yes/no), total energy intake, intake of vegetables, fruits, dairy food and alcohol consumption categories (zero/low/high). Model 3 further adjusted for coffee consumption. HDL, high-density-lipoprotein-cholesterol.

b *P* value associated with the HR.

c *P*_trend_ was obtained by using the median values of each consumption group as the exposure variables in the Cox proportional hazard models.

d Due to the violation of proportional hazard assumption, vegetable intake was replaced by the interaction term between vegetable intake and time in models 2 and 3.

### Sensitivity analysis

Excluding participants reported consuming decaffeinated coffee at baseline did not appreciably change the result of the main analysis (result not shown). Association between coffee intake and metabolic syndrome was not clinically and statistically significant when restricted to tea abstainers (Supplementary Table S1^3^). The same was observed for the association between tea intake and metabolic syndrome when restricted to coffee abstainers (Supplementary Table S1^4^).

No significant interaction was found in the association between coffee consumption and metabolic syndrome, as well as other component conditions. When modelling the association between tea consumption and metabolic syndrome incidence, a significant interaction was found between fruit intake and tea consumption (*P*_interaction_ = 0⋅007). After adjusting for all other covariates, participants with a higher fruit intake and consumed 4 cups of tea per day were inversely associated with the risk of metabolic syndrome when compared with those at the lowest consumption category, yet the association was not statistically significant (Table 5, multivariate-adjusted HR: 0⋅44, 95 % CI 0⋅20, 0⋅93, *P* = 0⋅033). No significant associations were found among participants with lower fruit intake. No significant interactions were found for other covariates.

**Table 5.** The association between tea consumption and metabolic syndrome, stratified according to fruit intake^a^

	1 cup/d or less	2–3 cups/d		4 cups/d or more		
	HR	HR (95 % CI)	*P*^b^	HR (95 % CI)	*P*^b^	*P*_trend_^c^
Low fruit intake (mean intake ± sd: 166 ± 82 g)						
No. of case/total no. of participants, *n*/total *n*	24/273	17/254		30/235		
Model 1	1 (ref)	0⋅79 (0⋅42, 1⋅47)	0⋅458	1⋅44 (0⋅84, 2⋅47)	0⋅189	0⋅265
Model 2	1 (ref)	0⋅74 (0⋅39, 1⋅41)	0⋅359	1⋅34 (0⋅77, 2⋅35)	0⋅303	0⋅391
Model 3	1 (ref)	0⋅79 (0⋅41, 1⋅49)	0⋅461	1⋅53 (0⋅85, 2⋅73)	0⋅154	0⋅227
High fruit intake (mean intake ± sd: 527 ± 222 g)						
No. of case/total no. of participants, *n*/total *n*	24/260	24/266		10/236		
Model 1	1 (ref)	1⋅09 (0⋅62, 1⋅94)	0⋅760	0⋅47 (0⋅22, 0⋅98)	0⋅043	0⋅093
Model 2	1 (ref)	1⋅08 (0⋅61, 1⋅93)	0⋅794	0⋅47 (0⋅22, 0⋅99)	0⋅046	0⋅098
Model 3	1 (ref)	1⋅07 (0⋅60, 1⋅91)	0⋅820	0⋅44 (0⋅20, 0⋅93)	0⋅033	0⋅078

a Stratification was carried out by conducting a median split on fruit intake, then analysing participants with high and low fruit intake separately. Model 1 adjusted for baseline age and sex. Model 2 further adjusted for smoker status (non-smoker/former smoker/current smoker), metabolic equivalents categories (zero/low/high), qualification obtained after leaving school (yes/no), family history of type 2 diabetes mellitus (yes/no), total energy intake, intake of vegetables, fruits, dairy food and alcohol consumption categories (zero/low/high). Model 3 further adjusted for coffee consumption.

b *P* value associated with the HR.

c *P*_trend_ was obtained by using the median values of each consumption group as the exposure variables in the Cox proportional hazard models.

## Discussion

In this longitudinal study that involved Australian older adults and a follow-up time of 10 years, tea and coffee consumption alone were not individually associated with the risk of having metabolic syndrome, high fasting glucose levels, high triglycerides levels, high BP and central obesity. Tea consumption at 2 cups/d or above, but not coffee consumption, could be associated with a lower risk of having low HDL-C. Consuming 4 cups of tea per day may also be associated with lower risks of metabolic syndrome in participants with high fruit intake.

One possible driver behind the inverse association between tea and fruit consumption and metabolic syndrome was the rich polyphenol content in both food items. Polyphenol intake was associated with lower metabolic syndrome prevalence^(37)^ and both tea and fruit were major contributors of polyphenol intake in the BMES cohort^(38)^. Polyphenols that are commonly present in both tea and fruit are mainly flavonoids, including flavan-3-ols (e.g. thearubigin and catechin) and proanthocyanidins^(38)^. Both classes of polyphenols have been shown to improve different aspects of metabolic syndrome^(39)^. The fact that the inverse association, albeit statistically insignificant, was only observed in those with both high tea and fruit consumption suggested there may be a synergistic effect between the two food items, or it could simply be the long-term effect of high polyphenol intake in general^(40)^.

The lack of a significant longitudinal association between coffee consumption and metabolic syndrome was also observed in previous prospective studies^(14–16)^. This also agrees with a recent meta-analysis^(13)^ which did not observe a significant association between coffee consumption and metabolic syndrome. The null findings in this regard also implied that the inverse associations between coffee consumption and T2DM^(41)^ and CVD incidence^(42)^ were not due to improvements in biomarkers, thereby strengthening the argument that residual confounding could be behind these results^(43)^.

The inverse association between tea consumption and the incidence of low HDL-C level agrees with the findings of a recent longitudinal study involving Chinese adults, which found that habitual tea consumption was associated with a slower rate of decrease in HDL-C when compared with non-consumers^(44)^. Nonetheless, this finding contradicts with results of previous randomised controlled trials which observed null effects regarding both green tea and black tea consumption on HDL-C^(45,46)^, thus hinting on the possibility that the protective effect could take time to build up. Since caffeine intake *per se* was unlikely to affect HDL-C^(47)^, the polyphenols in tea were thought to be responsible for this observation, possibly via the inhibition of lipid absorption at the small intestine^(48)^. However, it should be noted that our results did not achieve statistical significance under a conservative *P*-value threshold, thus these results should be verified in future longitudinal studies with greater sample sizes.

Although the risk of having metabolic syndrome is expected to increase with age as homeostatic control worsens^(49)^, the incidence of metabolic syndrome in this cohort (10 %) is lower than those (25–50 %) observed in previous cohorts involving participants of a similar age^(50,51)^, while a previous longitudinal study involving younger participants had a similar incidence rate^(16)^. One possible reason behind this observation was the differences in the definitions of metabolic syndrome adopted by previous studies. This study adopted the criteria published by the IDF in 2006^(22)^, which central obesity is essential for metabolic syndrome ascertainment. On the other hand, previous cohorts^(50,51)^ adopted definitions that only require the presence of any three out of the five component conditions of metabolic syndrome^(52,53)^, making it easier to ascertain cases. The sensitivity of metabolic syndrome incidence towards different criteria used should be verified in future longitudinal studies.

Strengths of the present study include its prospective design which enables the establishment of the temporal sequence of events, the long-term follow-up of a population with homogenous demographic characteristics, and the use of a validated FFQ for the collection of dietary data. On the other hand, we caution the readers to some limitations when interpreting our results. First, due to the observational nature of this study, the possibility of unadjusted confounders, such as the use of medication to treat high fasting glucose levels, the habit of adding milk and sugar into coffee, types of coffee and tea consumed, and genetic variations, affecting the results could not be excluded. Second, the small number of incident cases available for analysis might limit the power of the analysis to observe an association, a problem that also existed in previously published studies that analysed metabolic syndrome incidence using data from cohorts that were designed to look at other outcomes^(15,16)^. We proceeded with the analysis regardless to fill the gap in the current body of evidence regarding metabolic syndrome development in elderly population. The statistically significant findings of this study could be taken for their direction of effect, yet the magnitudes of the associations should be verified in future epidemiological studies with greater sample sizes and number of cases. Third, the accuracy of coffee intake data collected by FFQ could be affected by measurement error and recall bias. Nonetheless, self-reports of coffee intake frequency were found to be highly accurate and reproducible^(54)^. Fourth, the rate of dropout and portions of participants with missing data in exposure and outcome variables in this cohort are higher than those recorded in previous longitudinal studies^(14,16)^ and this may have introduced selection bias. However, the characteristics of participants that were loss to follow-up for each outcome were similar to those of the included participants. Finally, since the data in this study were collected from Australian older adults, generalisation of the findings of this study to populations of other age groups and ethnicity should be done with caution.

## Conclusion

In this prospective cohort study involving Australian older adults, coffee consumption was not associated with the risk of having metabolic syndrome, high fasting glucose, high triglycerides, central obesity, high blood pressure and low HDL-C. Tea consumption was also not associated with metabolic syndrome, high fasting glucose, high triglycerides, central obesity and high blood pressure. Conversely, tea consumption might be associated with a lower risk of having low HDL-C and, together with high fruit consumption, could be associated with a mild reduction in the risk of developing metabolic syndrome. While these findings should be verified in future epidemiological studies with greater sample sizes, they suggest a high exposure to dietary polyphenol may improve metabolic health.

## Supplementary material

For supplementary material accompanying this paper visit https://doi.org/10.1017/jns.2022.78. click here to view supplementary material
