# Supplementary material for: The longitudinal association between coffee and tea consumption and the risk of metabolic syndrome and its component conditions in an older adult population
Source: J Nutr Sci. 2022 Sep 21;11:e79. doi: 10.1017/jns.2022.78 (PMC9554431; doi:10.1017/jns.2022.78)
Supplement: Supplementary file 1 [file S2048679022000787sup001.docx]

Supplemental Table 1 Baseline characteristics of participants that were loss to follow-up in the analysis of metabolic syndrome, stratified by coffee consumption frequencies. ^1^

|  | Less than 1 cup/week (n = 222) | 1 cup/week - 1cup/day (n = 224) | 2 cups/day or above (n = 246) |
| --- | --- | --- | --- |
| Age (years) | 70.5 ± 10.4 | 68.2 ± 10.5 | 65.1 ± 10.0 |
| Male (%) | 56.3 | 54.5 | 52.0 |
| Female (%) | 43.7 | 45.5 | 48.0 |
| Non-smoker (%) | 50.9 | 50.0 | 29.3 |
| Former smoker (%) | 39.6 | 34.4 | 44.7 |
| Current smoker (%) | 9.5 | 15.6 | 26.0 |
| Obtained qualification after leaving school (%) | 46.4 | 41.5 | 43.9 |
| *METs categories (%)* | | | |
| Zero METs | 40.5 | 32.1 | 26.8 |
| Low METs | 27.5 | 37.9 | 37.0 |
| High METs | 32.0 | 29.9 | 36.2 |
| With family history of T2DM (%) | 21.2 | 13.4 | 19.5 |
| Energy (kJ) | 8082 ± 2243 | 8428 ± 2153 | 8251 ± 2175 |
| *Alcohol consumption categories (%)* ^2^ | | | |
| Zero | 36.9 | 19.2 | 14.6 |
| Low | 36.0 | 37.5 | 32.5 |
| High | 27.0 | 43.3 | 52.8 |
| Vegetables (g) | 423 ± 197 | 428 ± 189 | 413 ± 180 |
| Fruit (g) | 331 ± 268 | 327 ± 227 | 290 ± 214 |
| Dairy (g) | 309 ± 237 | 322 ± 233 | 318 ± 257 |
| *Tea consumption categories (%)* | | | |
| 1 cup/day or less | 27.9 | 33.0 | 51.6 |
| 2-3 cups/day | 32.4 | 32.1 | 34.1 |
| 4 cups/day or above | 39.6 | 34.8 | 14.2 |

^1^ Values are mean ± SD or percentages. METs, metabolic equivalents. T2DM, type 2 diabetes mellitus.

^2^ Participants in the “zero” category reported zero alcohol consumption. The mean ± SD of alcohol consumption in the low and high categories were 2 ± 2 g and 25 ± 18 g respectively.

Supplemental Table 2 Baseline characteristics of participants that were loss to follow-up in the analysis of high fasting glucose, stratified by coffee consumption frequencies. ^1^

|  | Less than 1 cup/week (n = 207) | 1 cup/week - 1cup/day (n = 221) | 2 cups/day or above (n = 244) |
| --- | --- | --- | --- |
| Age (years) | 69.5 ± 10.3 | 66.6 ± 10.1 | 64.5 ± 10.1 |
| Male (%) | 58.0 | 58.8 | 56.6 |
| Female (%) | 42.0 | 41.2 | 43.4 |
| Non-smoker (%) | 46.9 | 51.6 | 32.8 |
| Former smoker (%) | 43.5 | 34.8 | 39.3 |
| Current smoker (%) | 9.7 | 13.6 | 27.9 |
| Obtained qualification after leaving school (%) | 45.9 | 38.0 | 48.0 |
| *METs categories (%)* | | | |
| Zero METs | 30.9 | 29.4 | 27.9 |
| Low METs | 31.9 | 39.8 | 36.9 |
| High METs | 37.2 | 30.8 | 35.2 |
| With family history of T2DM (%) | 19.3 | 12.7 | 19.3 |
| Energy (kJ) | 8186 ± 2175 | 8271 ± 2163 | 8328 ± 2210 |
| *Alcohol consumption categories (%)* ^2^ | | | |
| Zero | 32.9 | 19.0 | 16.8 |
| Low | 38.6 | 38.5 | 31.6 |
| High | 28.5 | 42.5 | 51.6 |
| Vegetables (g) | 424 ± 182 | 431 ± 189 | 417 ± 173 |
| Fruit (g) | 321 ± 266 | 326 ± 234 | 309 ± 238 |
| Dairy (g) | 340 ± 260 | 323 ± 230 | 317 ± 257 |
| *Tea consumption categories (%)* | | | |
| 1 cup/day or less | 23.2 | 30.8 | 52.5 |
| 2-3 cups/day | 35.3 | 33.9 | 33.2 |
| 4 cups/day or above | 41.5 | 35.3 | 14.3 |

^1^ Values are mean ± SD or percentages. METs, metabolic equivalents. T2DM, type 2 diabetes mellitus.

^2^ Participants in the “zero” category reported zero alcohol consumption. The mean ± SD of alcohol consumption in the low and high categories were 2 ± 2 g and 25 ± 18 g respectively.

Supplemental Table 3 Baseline characteristics of participants that were loss to follow-up in the analysis of high triglycerides, stratified by coffee consumption frequencies. ^1^

|  | Less than 1 cup/week (n = 146) | 1 cup/week - 1cup/day (n = 164) | 2 cups/day or above (n = 181) |
| --- | --- | --- | --- |
| Age (years) | 70.8 ± 10.6 | 68.0 ± 10.6 | 65.8 ± 10.3 |
| Male (%) | 52.7 | 59.1 | 58.0 |
| Female (%) | 47.3 | 40.9 | 42.0 |
| Non-smoker (%) | 51.4 | 56.1 | 32.6 |
| Former smoker (%) | 42.5 | 35.4 | 39.8 |
| Current smoker (%) | 6.2 | 8.5 | 27.6 |
| Obtained qualification after leaving school (%) | 46.6 | 37.8 | 49.2 |
| *METs categories (%)* | | | |
| Zero METs | 30.1 | 31.1 | 29.8 |
| Low METs | 34.2 | 37.2 | 39.2 |
| High METs | 35.6 | 31.7 | 30.9 |
| With family history of T2DM (%) | 21.2 | 14.6 | 18.8 |
| Energy (kJ) | 8293 ± 2285 | 8165 ± 2165 | 8078 ± 2054 |
| *Alcohol consumption categories (%)* ^2^ | | | |
| Zero | 32.9 | 22.0 | 18.8 |
| Low | 37.0 | 36.6 | 33.1 |
| High | 30.1 | 41.5 | 48.1 |
| Vegetables (g) | 424 ± 194 | 434 ± 193 | 410 ± 172 |
| Fruit (g) | 311 ± 283 | 326 ± 222 | 321 ± 245 |
| Dairy (g) | 325 ± 257 | 311 ± 203 | 338 ± 276 |
| *Tea consumption categories (%)* | | | |
| 1 cup/day or less | 28.8 | 29.3 | 50.8 |
| 2-3 cups/day | 34.2 | 29.9 | 33.1 |
| 4 cups/day or above | 37.0 | 40.9 | 16.0 |

^1^ Values are mean ± SD or percentages. METs, metabolic equivalents. T2DM, type 2 diabetes mellitus.

^2^ Participants in the “zero” category reported zero alcohol consumption. The mean ± SD of alcohol consumption in the low and high categories were 2 ± 2 g and 25 ± 18 g respectively.

Supplemental Table 4 Baseline characteristics of participants that were loss to follow-up in the analysis of central obesity, stratified by coffee consumption frequencies. ^1^

|  | Less than 1 cup/week (n = 207) | 1 cup/week - 1cup/day (n = 195) | 2 cups/day or above (n = 223) |
| --- | --- | --- | --- |
| Age (years) | 71.1 ± 10.4 | 69.1 ± 10.3 | 65.4 ± 10.2 |
| Male (%) | 57.0 | 53.8 | 50.2 |
| Female (%) | 43.0 | 46.2 | 49.8 |
| Non-smoker (%) | 51.7 | 48.2 | 27.8 |
| Former smoker (%) | 38.2 | 33.8 | 44.8 |
| Current smoker (%) | 10.1 | 17.9 | 27.4 |
| Obtained qualification after leaving school (%) | 47.8 | 41.5 | 44.4 |
| *METs categories (%)* | | | |
| Zero METs | 42.5 | 30.8 | 26.0 |
| Low METs | 28.0 | 39.0 | 36.8 |
| High METs | 29.5 | 30.3 | 37.2 |
| With family history of T2DM (%) | 19.3 | 13.8 | 20.2 |
| Energy (kJ) | 8051 ± 2208 | 8396 ± 2193 | 8327 ± 2148 |
| *Alcohol consumption categories (%)* ^2^ | | | |
| Zero | 37.2 | 20.0 | 13.0 |
| Low | 36.7 | 37.4 | 33.2 |
| High | 26.1 | 42.6 | 53.8 |
| Vegetables (g) | 422 ± 199 | 431 ± 193 | 416 ± 180 |
| Fruit (g) | 327 ± 269 | 315 ± 217 | 289 ± 217 |
| Dairy (g) | 303 ± 231 | 313 ± 226 | 312 ± 251 |
| *Tea consumption categories (%)* | | | |
| 1 cup/day or less | 28.0 | 32.8 | 52.9 |
| 2-3 cups/day | 32.4 | 30.3 | 33.2 |
| 4 cups/day or above | 39.6 | 36.9 | 13.9 |

^1^ Values are mean ± SD or percentages. METs, metabolic equivalents. T2DM, type 2 diabetes mellitus.

^2^ Participants in the “zero” category reported zero alcohol consumption. The mean ± SD of alcohol consumption in the low and high categories were 2 ± 2 g and 25 ± 18 g respectively.

Supplemental Table 5 Baseline characteristics of participants that were loss to follow-up in the analysis of high blood pressure, stratified by coffee consumption frequencies. ^1^

|  | Less than 1 cup/week (n = 34) | 1 cup/week - 1cup/day (n = 27) | 2 cups/day or above (n = 40) |
| --- | --- | --- | --- |
| Age (years) | 67.0 ± 11.2 | 62.2 ± 11.8 | 60.1 ± 8.4 |
| Male (%) | 44.1 | 55.6 | 50.0 |
| Female (%) | 55.9 | 44.4 | 50.0 |
| Non-smoker (%) | 50.0 | 51.9 | 25.0 |
| Former smoker (%) | 41.2 | 33.3 | 35.0 |
| Current smoker (%) | 8.8 | 14.8 | 40.0 |
| Obtained qualification after leaving school (%) | 38.2 | 29.6 | 37.5 |
| *METs categories (%)* | | | |
| Zero METs | 32.4 | 33.3 | 32.5 |
| Low METs | 32.4 | 37.0 | 37.5 |
| High METs | 35.3 | 29.6 | 30.0 |
| With family history of T2DM (%) | 20.6 | 22.2 | 35.0 |
| Energy (kJ) | 7415 ± 2042 | 8190 ± 2297 | 8376 ± 2061 |
| *Alcohol consumption categories (%)* ^2^ | | | |
| Zero | 41.2 | 14.8 | 22.5 |
| Low | 26.5 | 51.9 | 30.0 |
| High | 32.4 | 33.3 | 47.5 |
| Vegetables (g) | 358 ± 126 | 386 ± 213 | 384 ± 181 |
| Fruit (g) | 287 ± 212 | 289 ± 185 | 280 ± 208 |
| Dairy (g) | 269 ± 238 | 353 ± 276 | 287 ± 217 |
| *Tea consumption categories (%)* | | | |
| 1 cup/day or less | 41.2 | 48.1 | 65.0 |
| 2-3 cups/day | 26.5 | 33.3 | 25.0 |
| 4 cups/day or above | 32.4 | 18.5 | 10.0 |

^1^ Values are mean ± SD or percentages. METs, metabolic equivalents. T2DM, type 2 diabetes mellitus.

^2^ Participants in the “zero” category reported zero alcohol consumption. The mean ± SD of alcohol consumption in the low and high categories were 2 ± 2 g and 25 ± 18 g respectively.

Supplemental Table 6 Baseline characteristics of participants that were loss to follow-up in the analysis of low HDL-cholesterol, stratified by coffee consumption frequencies. ^1^

|  | Less than 1 cup/week (n = 187) | 1 cup/week - 1cup/day (n = 203) | 2 cups/day or above (n = 211) |
| --- | --- | --- | --- |
| Age (years) | 69.8 ± 10.6 | 67.4 ± 10.5 | 65.2 ± 10.2 |
| Male (%) | 73.8 | 72.4 | 74.9 |
| Female (%) | 26.2 | 27.6 | 25.1 |
| Non-smoker (%) | 51.9 | 54.2 | 35.5 |
| Former smoker (%) | 38.5 | 31.5 | 37.4 |
| Current smoker (%) | 9.6 | 14.3 | 27.0 |
| Obtained qualification after leaving school (%) | 48.1 | 36.9 | 48.3 |
| *METs categories (%)* | | | |
| Zero METs | 33.2 | 33.0 | 28.4 |
| Low METs | 33.2 | 36.0 | 40.3 |
| High METs | 33.7 | 31.0 | 31.3 |
| With family history of T2DM (%) | 21.4 | 15.8 | 18.5 |
| Energy (kJ) | 8127 ± 2217 | 7975 ± 2072 | 8090 ± 2078 |
| *Alcohol consumption categories (%)* ^2^ | | | |
| Zero | 34.2 | 22.7 | 19.0 |
| Low | 40.1 | 37.4 | 33.6 |
| High | 25.7 | 39.9 | 47.4 |
| Vegetables (g) | 439 ± 205 | 438 ± 196 | 427 ± 185 |
| Fruit (g) | 329 ± 272 | 326 ± 228 | 325 ± 259 |
| Dairy (g) | 337 ± 255 | 314 ± 215 | 323 ± 258 |
| *Tea consumption categories (%)* | | | |
| 1 cup/day or less | 28.9 | 32.5 | 53.6 |
| 2-3 cups/day | 33.7 | 32.5 | 31.8 |
| 4 cups/day or above | 37.4 | 35.0 | 14.7 |

^1^ Values are mean ± SD or percentages. METs, metabolic equivalents. T2DM, type 2 diabetes mellitus. HDL-C, high-density-lipoprotein cholesterol.

^2^ Participants in the “zero” category reported zero alcohol consumption. The mean ± SD of alcohol consumption in the low and high categories were 2 ± 2 g and 25 ± 18 g respectively.

Supplemental Table 7 Baseline characteristics of participants that were loss to follow-up in the analysis of metabolic syndrome, stratified by tea consumption frequencies. ^1^

|  | 1 cup/day or less (n = 263) | 2-3 cups/day (n = 228) | 4 cups/day or above (n = 201) |
| --- | --- | --- | --- |
| Age (years) | 66.5 ± 10.4 | 68.2 ± 10.9 | 69.3 ± 10.0 |
| Male (%) | 54.0 | 50.9 | 58.2 |
| Female (%) | 46.0 | 49.1 | 41.8 |
| Non-smoker (%) | 38.8 | 46.9 | 43.8 |
| Former smoker (%) | 41.1 | 37.3 | 40.8 |
| Current smoker (%) | 20.2 | 15.8 | 15.4 |
| Obtained qualification after leaving school (%) | 43.0 | 42.1 | 47.3 |
| *METs categories (%)* | | | |
| Zero METs | 33.8 | 31.6 | 33.3 |
| Low METs | 31.2 | 33.8 | 38.8 |
| High METs | 35.0 | 34.6 | 27.9 |
| With family history of T2DM (%) | 17.1 | 17.1 | 20.4 |
| Energy (kJ) | 7869 ± 2214 | 8323 ± 2272 | 8680 ± 1982 |
| *Alcohol consumption categories (%)* ^2^ | | | |
| Zero | 22.1 | 18.9 | 29.9 |
| Low | 32.3 | 36.4 | 37.8 |
| High | 45.6 | 44.7 | 32.3 |
| Vegetables (g) | 402 ± 201 | 421 ± 183 | 447 ± 175 |
| Fruit (g) | 316 ± 251 | 312 ± 220 | 318 ± 238 |
| Dairy (g) | 284 ± 245 | 326 ± 255 | 348 ± 220 |
| *Coffee consumption categories (%)* | | | |
| Less than 1 cup/week | 23.6 | 31.6 | 43.8 |
| 1 cup/week – 1 cup/day | 28.1 | 31.6 | 38.8 |
| 2 cups/day or above | 48.3 | 36.8 | 17.4 |

^1^ Values are mean ± SD or percentages. METs, metabolic equivalents. T2DM, type 2 diabetes mellitus.

^2^ Participants in the “zero” category reported zero alcohol consumption. The mean ± SD of alcohol consumption in the low and high categories were 2 ± 2 g and 25 ± 18 g respectively.

Supplemental Table 8 Baseline characteristics of participants that were loss to follow-up in the analysis of high fasting glucose, stratified by tea consumption frequencies. ^1^

|  | 1 cup/day or less (n = 244) | 2-3 cups/day (n = 229) | 4 cups/day or above (n = 199) |
| --- | --- | --- | --- |
| Age (years) | 65.1 ± 10.1 | 67.4 ± 10.8 | 68.0 ± 9.9 |
| Male (%) | 58.6 | 54.6 | 60.3 |
| Female (%) | 41.4 | 45.4 | 39.7 |
| Non-smoker (%) | 38.1 | 47.6 | 44.7 |
| Former smoker (%) | 37.7 | 39.7 | 40.2 |
| Current smoker (%) | 24.2 | 12.7 | 15.1 |
| Obtained qualification after leaving school (%) | 44.7 | 45.4 | 41.7 |
| *METs categories (%)* | | | |
| Zero METs | 32.4 | 29.3 | 25.6 |
| Low METs | 32.8 | 34.1 | 43.2 |
| High METs | 34.8 | 36.7 | 31.2 |
| With family history of T2DM (%) | 17.2 | 16.6 | 17.6 |
| Energy (kJ) | 7885 ± 2236 | 8390 ± 2185 | 8588 ± 2047 |
| *Alcohol consumption categories (%)* ^2^ | | | |
| Zero | 23.0 | 18.8 | 26.1 |
| Low | 29.5 | 39.3 | 40.2 |
| High | 47.5 | 41.9 | 33.7 |
| Vegetables (g) | 409 ± 186 | 426 ± 186 | 439 ± 168 |
| Fruit (g) | 319 ± 258 | 314 ± 234 | 321 ± 243 |
| Dairy (g) | 282 ± 251 | 338 ± 258 | 366 ± 229 |
| *Coffee consumption categories (%)* | | | |
| Less than 1 cup/week | 19.7 | 31.9 | 43.2 |
| 1 cup/week – 1 cup/day | 27.9 | 32.8 | 39.2 |
| 2 cups/day or above | 52.5 | 35.4 | 17.6 |

^1^ Values are mean ± SD or percentages. METs, metabolic equivalents. T2DM, type 2 diabetes mellitus.

^2^ Participants in the “zero” category reported zero alcohol consumption. The mean ± SD of alcohol consumption in the low and high categories were 2 ± 2 g and 25 ± 18 g respectively.

Supplemental Table 9 Baseline characteristics of participants that were loss to follow-up in the analysis of high triglycerides, stratified by tea consumption frequencies. ^1^

|  | 1 cup/day or less (n = 182) | 2-3 cups/day (n = 159) | 4 cups/day or above (n = 150) |
| --- | --- | --- | --- |
| Age (years) | 66.2 ± 10.8 | 69.4 ± 10.8 | 68.7 ± 10.1 |
| Male (%) | 56.0 | 52.8 | 62.0 |
| Female (%) | 44.0 | 47.2 | 38.0 |
| Non-smoker (%) | 40.7 | 50.9 | 47.3 |
| Former smoker (%) | 36.8 | 38.4 | 42.7 |
| Current smoker (%) | 22.5 | 10.7 | 10.0 |
| Obtained qualification after leaving school (%) | 47.8 | 45.3 | 40.0 |
| *METs categories (%)* | | | |
| Zero METs | 34.1 | 28.3 | 28.0 |
| Low METs | 33.5 | 35.8 | 42.7 |
| High METs | 32.4 | 35.8 | 29.3 |
| With family history of T2DM (%) | 19.2 | 17.6 | 17.3 |
| Energy (kJ) | 7592 ± 2109 | 8453 ± 2213 | 8573 ± 2018 |
| *Alcohol consumption categories (%)* ^2^ | | | |
| Zero | 24.7 | 21.4 | 26.0 |
| Low | 29.7 | 37.1 | 40.7 |
| High | 45.6 | 41.5 | 33.3 |
| Vegetables (g) | 399 ± 180 | 437 ± 198 | 434 ± 176 |
| Fruit (g) | 324 ± 265 | 313 ± 233 | 321 ± 248 |
| Dairy (g) | 280 ± 255 | 345 ± 250 | 358 ± 228 |
| *Coffee consumption categories (%)* | | | |
| Less than 1 cup/week | 23.1 | 31.4 | 36.0 |
| 1 cup/week – 1 cup/day | 26.4 | 30.8 | 44.7 |
| 2 cups/day or above | 50.5 | 37.7 | 19.3 |

^1^ Values are mean ± SD or percentages. METs, metabolic equivalents. T2DM, type 2 diabetes mellitus.

^2^ Participants in the “zero” category reported zero alcohol consumption. The mean ± SD of alcohol consumption in the low and high categories were 2 ± 2 g and 25 ± 18 g respectively.

Supplemental Table 10 Baseline characteristics of participants that were loss to follow-up in the analysis of central obesity, stratified by tea consumption frequencies. ^1^

|  | 1 cup/day or less (n = 240) | 2-3 cups/day (n = 200) | 4 cups/day or above (n = 185) |
| --- | --- | --- | --- |
| Age (years) | 66.8 ± 10.6 | 68.9 ± 10.8 | 70.1 ± 9.9 |
| Male (%) | 52.5 | 51.5 | 57.3 |
| Female (%) | 47.5 | 48.5 | 42.7 |
| Non-smoker (%) | 37.9 | 46.5 | 42.7 |
| Former smoker (%) | 41.2 | 35.5 | 40.5 |
| Current smoker (%) | 20.8 | 18.0 | 16.8 |
| Obtained qualification after leaving school (%) | 44.2 | 42.0 | 48.1 |
| *METs categories (%)* | | | |
| Zero METs | 33.3 | 31.0 | 34.6 |
| Low METs | 31.7 | 33.0 | 40.0 |
| High METs | 35.0 | 36.0 | 25.4 |
| With family history of T2DM (%) | 17.9 | 16.5 | 19.5 |
| Energy (kJ) | 7880 ± 2183 | 8354 ± 2283 | 8641 ± 1999 |
| *Alcohol consumption categories (%)* ^2^ | | | |
| Zero | 21.7 | 18.5 | 30.3 |
| Low | 32.5 | 36.0 | 39.5 |
| High | 45.8 | 45.5 | 30.3 |
| Vegetables (g) | 406 ± 203 | 425 ± 188 | 443 ± 174 |
| Fruit (g) | 316 ± 258 | 300 ± 203 | 312 ± 240 |
| Dairy (g) | 278 ± 238 | 317 ± 253 | 341 ± 211 |
| *Coffee consumption categories (%)* | | | |
| Less than 1 cup/week | 24.2 | 33.5 | 44.3 |
| 1 cup/week – 1 cup/day | 26.7 | 29.5 | 38.9 |
| 2 cups/day or above | 49.2 | 37.0 | 16.8 |

^1^ Values are mean ± SD or percentages. METs, metabolic equivalents. T2DM, type 2 diabetes mellitus.

^2^ Participants in the “zero” category reported zero alcohol consumption. The mean ± SD of alcohol consumption in the low and high categories were 2 ± 2 g and 25 ± 18 g respectively.

Supplemental Table 11 Baseline characteristics of participants that were loss to follow-up in the analysis of high blood pressure, stratified by tea consumption frequencies. ^1^

|  | 1 cup/day or less (n = 53) | 2-3 cups/day (n = 28) | 4 cups/day or above (n = 20) |
| --- | --- | --- | --- |
| Age (years) | 61.7 ± 9.1 | 63.9 ± 12.2 | 65.3 ± 12.3 |
| Male (%) | 60.4 | 32.1 | 45.0 |
| Female (%) | 39.6 | 67.9 | 55.0 |
| Non-smoker (%) | 39.6 | 42.9 | 40.0 |
| Former smoker (%) | 41.5 | 32.1 | 30.0 |
| Current smoker (%) | 18.9 | 25.0 | 30.0 |
| Obtained qualification after leaving school (%) | 34.0 | 42.9 | 30.0 |
| *METs categories (%)* | | | |
| Zero METs | 41.5 | 14.3 | 35.0 |
| Low METs | 30.2 | 35.7 | 50.0 |
| High METs | 28.3 | 50.0 | 15.0 |
| With family history of T2DM (%) | 30.2 | 17.9 | 30.0 |
| Energy (kJ) | 7506 ± 1927 | 8775 ± 2296 | 8236 ± 2206 |
| *Alcohol consumption categories (%)* ^2^ | | | |
| Zero | 32.1 | 21.4 | 20.0 |
| Low | 32.1 | 35.7 | 40.0 |
| High | 35.8 | 42.9 | 40.0 |
| Vegetables (g) | 370 ± 203 | 354 ± 127 | 420 ± 140 |
| Fruit (g) | 309 ± 207 | 251 ± 164 | 268 ± 235 |
| Dairy (g) | 255 ± 179 | 404 ± 348 | 267 ± 153 |
| *Coffee consumption categories (%)* | | | |
| Less than 1 cup/week | 26.4 | 32.1 | 55.0 |
| 1 cup/week – 1 cup/day | 24.5 | 32.1 | 25.0 |
| 2 cups/day or above | 49.1 | 35.7 | 20.0 |

^1^ Values are mean ± SD or percentages. METs, metabolic equivalents. T2DM, type 2 diabetes mellitus.

^2^ Participants in the “zero” category reported zero alcohol consumption. The mean ± SD of alcohol consumption in the low and high categories were 2 ± 2 g and 25 ± 18 g respectively.

Supplemental Table 12 Baseline characteristics of participants that were loss to follow-up in the analysis of low HDL-cholesterol, stratified by tea consumption frequencies. ^1^

|  | 1 cup/day or less (n = 233) | 2-3 cups/day (n = 196) | 4 cups/day or above (n = 172) |
| --- | --- | --- | --- |
| Age (years) | 65.8 ± 10.5 | 68.0 ± 10.7 | 68.8 ± 10.2 |
| Male (%) | 71.7 | 70.9 | 79.7 |
| Female (%) | 28.3 | 29.1 | 20.3 |
| Non-smoker (%) | 41.6 | 51.5 | 48.8 |
| Former smoker (%) | 35.6 | 34.7 | 37.2 |
| Current smoker (%) | 22.7 | 13.8 | 14.0 |
| Obtained qualification after leaving school (%) | 46.8 | 42.3 | 43.6 |
| *METs categories (%)* | | | |
| Zero METs | 33.0 | 29.1 | 32.0 |
| Low METs | 33.0 | 37.2 | 40.7 |
| High METs | 33.9 | 33.7 | 27.3 |
| With family history of T2DM (%) | 18.9 | 17.3 | 19.2 |
| Energy (kJ) | 7733 ± 2134 | 8073 ± 2089 | 8499 ± 2058 |
| *Alcohol consumption categories (%)* ^2^ | | | |
| Zero | 24.5 | 20.9 | 30.2 |
| Low | 32.2 | 39.8 | 40.1 |
| High | 43.3 | 39.3 | 29.7 |
| Vegetables (g) | 418 ± 195 | 433 ± 193 | 459 ± 194 |
| Fruit (g) | 336 ± 272 | 311 ± 235 | 332 ± 245 |
| Dairy (g) | 279 ± 243 | 338 ± 241 | 370 ± 236 |
| *Coffee consumption categories (%)* | | | |
| Less than 1 cup/week | 23.2 | 32.1 | 40.7 |
| 1 cup/week – 1 cup/day | 28.3 | 33.7 | 41.3 |
| 2 cups/day or above | 48.5 | 34.2 | 18.0 |

^1^ Values are mean ± SD or percentages. METs, metabolic equivalents. T2DM, type 2 diabetes mellitus. HDL-C, high-density-lipoprotein cholesterol.

^2^ Participants in the “zero” category reported zero alcohol consumption. The mean ± SD of alcohol consumption in the low and high categories were 2 ± 2 g and 25 ± 18 g respectively.

Supplementary table 13 The association between coffee consumption frequency and metabolic syndrome among tea abstainers only. ^1^

|  | Less than 1 cup/week | 1 cup/week – 1 cup/day | | 2 cups/day or more | |  |
| --- | --- | --- | --- | --- | --- | --- |
|  | HR | HR (95% CI) | *P ^2^* | HR (95% CI) | *P ^2^* | *P _trend_ ^3^* |
| no. of case/ total no. of participants, *n*/total *n* | 2/46 | 3/23 |  | 10/110 |  |  |
| Model 1 | 1 (ref) | 3.15 (0.52, 19.07) | 0.213 | 1.98 (0.43, 9.08) | 0.381 | 0.549 |
| Model 2 | 1 (ref) | 4.29 (0.60, 30.82) | 0.147 | 2.17 (0.45, 10.40) | 0.332 | 0.550 |

^1^ Model 1 adjusted for baseline age and sex. Model 2 further adjusted for smoker status (non-smoker/former smoker/current smoker), metabolic equivalents categories (zero/low/high), qualification obtained after leaving school (yes/no), family history of type 2 diabetes mellitus (yes/no), total energy intake, intake of vegetables, fruits, dairy food, and alcohol consumption categories (zero/low/high).

^2^ *P*-value associated with the HR.

^3^ *P _trend_* was obtained by using the median values of each consumption group as the exposure variables in the Cox proportional hazard models.

Supplementary table 14 The association between tea consumption frequency and metabolic syndrome among coffee abstainers only. ^1^

|  | 1 cup/day or less | 2-3 cups/day | | 4 cups/day or more | |  |
| --- | --- | --- | --- | --- | --- | --- |
|  | HR | HR (95% CI) | *P ^2^* | HR (95% CI) | *P ^2^* | *P _trend_ ^3^* |
| no. of case/ total no. of participants, *n*/total *n* | 5/75 | 4/62 |  | 4/88 |  |  |
| Model 1 | 1 (ref) | 1.28 (0.34, 4.84) | 0.717 | 0.63 (0.17, 2.36) | 0.494 | 0.553 |
| Model 2 | 1 (ref) | 1.39 (0.31, 6.24) | 0.667 | 1.17 (0.22, 6.35) | 0.851 | 0.787 |

^1^ Model 1 adjusted for baseline age and sex. Model 2 further adjusted for smoker status (non-smoker/former smoker/current smoker), metabolic equivalents categories (zero/low/high), qualification obtained after leaving school (yes/no), family history of type 2 diabetes mellitus (yes/no), total energy intake, intake of vegetables, fruits, dairy food, and alcohol consumption categories (zero/low/high).

^2^ *P*-value associated with the HR.

^3^ *P _trend_* was obtained by using the median values of each consumption group as the exposure variables in the Cox proportional hazard models.
